# Wide-area low-energy surface stimulation of large mammalian ventricular tissue

**DOI:** 10.1038/s41598-019-51364-w

**Published:** 2019-11-01

**Authors:** Angel Moreno, Richard D. Walton, Marion Constantin, Olivier Bernus, Edward J. Vigmond, Jason D. Bayer

**Affiliations:** 1 IHU-LIRYC, Electrophysiology and Heart Modeling Institute, Fondation Bordeaux Université. Pessac, Bordeaux, France; 2 Centre National De La Recherche Scientifique, Institut de Mathématiques de Bordeaux, UMR5251 Bordeaux, France; 3 Centre de Recherche Cardio-Thoracique de Bordeaux, Université de Bordeaux, U1045 Bordeaux, France; 4 INSERM, Centre de recherche Cardio-Thoracique de Bordeaux, U1045 Bordeaux, France; 5 Present Address: L’Institut de Rythmologie et Modélisation Cardiaque (LIRYC), Hôpital Xavier Arnozan, Avenue du Haut Lévêque, 33604 Pessac, France

**Keywords:** Cardiology, Biomedical engineering

## Abstract

The epicardial and endocardial surfaces of the heart are attractive targets to administer antiarrhythmic electrotherapies. Electrically stimulating wide areas of the surfaces of small mammalian ventricles is straightforward given the relatively small scale of their myocardial dimensions compared to the tissue space constant and electrical field. However, it has yet to be proven for larger mammalian hearts with tissue properties and ventricular dimensions closer to humans. Our goal was to address the feasibility and impact of wide-area electrical stimulation on the ventricular surfaces of large mammalian hearts at different stimulus strengths. This was accomplished by placing long line electrodes on the ventricular surfaces of pig hearts that span wide areas, and activating them individually. Stimulus efficacy was assessed and compared between surfaces, and tissue viability was evaluated. Activation time was dependent on stimulation strength and location, achieving uniform linear stimulation at 9x threshold strength. Endocardial stimulation activated more tissue transmurally than epicardial stimulation, which could be considered a potential target for future cardiac electrotherapies. Overall, our results indicate that electrically stimulating wide areas of the ventricular surfaces of large mammals is achievable with line electrodes, minimal tissue damage, and energies under the human pain threshold (100 mJ).

## Introduction

The epicardial and endocardial surfaces of the heart are attractive targets to administer antiarrhythmic electrotherapies, especially in patients with higher risks of sudden cardiac death due to serious arrhythmias such as ventricular tachycardia (VT) and ventricular fibrillation (VF). Tradionally, implantable cardiac electrotherapy devices comprise a maximum of 3 stimulating electrodes. Consequently, this limits the spatial coverage for targeting specific regions of the heart and leads to higher energy cardiac electrotherapies to compensate for this. As an alternative, stretchable electronics are capable of being applied directly to the epicardial surface of mammalian ventricles^1,2^, which provides high spatial resolution, wide-area coverage, and versatility for cardiac monitoring and therapy. Although surface stimulation through conformal devices is encouraging, this technology has been primarily tested in small mammals, and its feasibility in large mammalian hearts with tissue properties and ventricular dimensions closer to humans has not yet been sufficiently established.

Electrically stimulating wide areas of large mammalian ventricles is more challenging than small mammals. Specifically, the ventricular volume of dogs, sheep, pigs, and humans is more than an order of magnitude greater than that of mice, rats, and rabbits^3^. Therefore, small mammals have less surface area, which makes wide-area electrical stimulation possible^1,4^ with less surface electrode contact compared to large mammals. Secondly, large mammalian ventricles have considerable spatial heterogeneity^5^. As a consequence, the threshold for stimulation is non-uniform across the ventricles and requires more current/energy to achieve simultaneous stimulation across all electrodes. Lastly, large mammals have a pronounced vascular network on the ventricular epicardium, as well as large papillary muscles and complex trabeculae on the endocardium. Thus, electrodes spanning wide areas have to be strategically placed on ventricular endocardial surfaces to obtain optimal electrical contact, while on the epicardium, special care is needed to not occlude or damage the important vascular network.

To circumvent these limitations, acute electrical field stimulation using mesh electrodes or pacing leads located on or in close proximity to the heart has been proposed to uniformly activate cardiac surfaces of large mammals^6–9^. Although useful for analyzing the effects of shock-induced stimulations deep in the tissue, it does not provide valuable insight into targeted stimulation of the heart by electrodes in direct contact with the different cardiac surfaces. Likewise, a set of wire electrodes (approximately 3.1 cm long) placed on the right ventricular canine epicardium has been suggested to uniformly stimulate cardiac tissue^10^; but its limited coverage area (approximately 3.1 × 3.1 cm) and stimulus performance do not directly assess the intrinsic heterogeneities over wide areas of the epicardium. To date, a systematic assessment of electrical stimulation strength, electrode positioning, and spacing is lacking to attain uniform stimulation across a wide-area spanning electrode. Also, it is not clear how stimulus efficacy is influenced by the stimulus location according to the cardiac surfaces (epicardium versus endocardium), and whether these stimulus strengths pose a risk to the tissue viability.

Accordingly, the main objective of this study was to address the feasibility and impact of wide-area electrical stimulation on the ventricular surfaces of large mammalian hearts. This investigation sought to specifically determine the following: (1) novel parameters for quantifying uniform stimulation around electrodes spanning wide areas of the heart, especially early activation parameters. Uniform stimulation across the line is of particular importance since it could potentially reduce the risk of forming new wave fronts that could initiate (or reinitiate) an arrhythmia^4^; (2) how these parameters change with respect to stimulus strength and the surface stimulated (endocardium versus epicardium); and (3) the safety of stimulus strength with respect to tissue and cellular viability.

To address these quetions, pig hearts were used due to their close resemblance to human heart size and electrophysiology^11^. Line electrodes were used as the conduits to deliver the electrical pulses at different strengths, and are ideal since they do not puncture the tissue, can adapt to the morphology of the heart, and are compatible with optical mapping techniques. Furthermore, *in silico* studies propose that the optimal electrode configuration to achieve a lower stimulation energy by wide-area stimulation is a series of evenly distributed lines, rather than a grid of lines or strategically placed points^12^. Thus, parallel lines presents the best tradeoff between surface area coverage and stimulus magnitude compared to the other configurations. Electrical responses of both cardiac surfaces were analyzed from optical mapping signals captured simultaneously, and activation time parameters were quantified and compared for the different stimulus strengths and locations.

## Results

**Figure 1:**
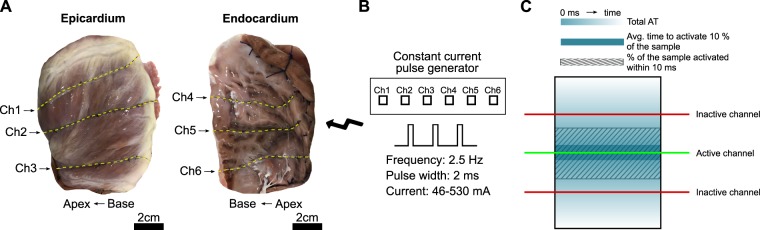
Overview of the implemented methods. (**A**) Three line electrodes were placed 2–3 cm apart on the epicardial and endocardial surfaces with an apico-basal orientation. (**B**) Each line electrode was connected to an isolated channel in the stimulator (constant current pulse generator) and stimulated with a frequency of 2.5 Hz, 2 ms pulse width, and current ranging from 46 to 530 mA. (**C**) The three main parameters computed when analyzing activation time (AT) with respect to the stimulated line (green line) were: Total AT, which was the elapsed time to activate all the tissue; average AT to activate 10% of the tissue, which corresponded to the area surrounding the line electrode that was being activated; and percentage of the tissue that was activated within 10 ms, which was from the onset of the stimulation.

**Figure 2:**
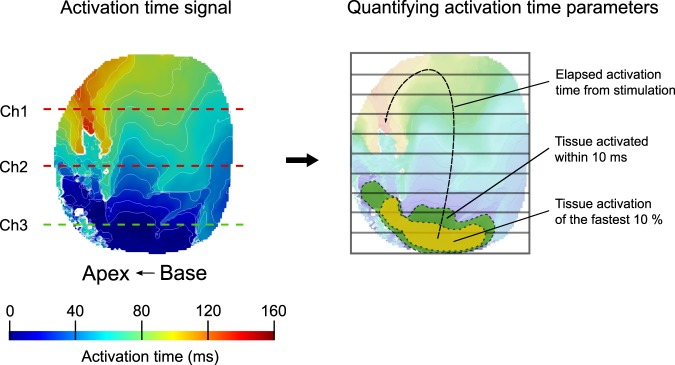
Quantification of activation time parameters. (Left) Representative epicardial AT signal during stimulation of channel 3 at 9x thr. Isochrone contour spacing was 12 ms. (Right) Quantification of the AT parameters performed on the original AT signal: tissue activation of the fastest 10% (yellow, near stimulus origin); and tissue that was activated within 10 ms (green) from the onset of the stimulation. Total AT was determined by the time it took the entire tissue to activate from the moment of the first activation, which was from the active line electrode in channel 3 (yellow and green area).

Pig hearts were stimulated at different strengths using a set of line electrodes placed on the epicardial and endocardial surfaces (Fig. 1), and signals were analyzed to assess activation performance (Fig. 2). Overall, a stronger stimulus strength yielded a uniform and linear response from the electrodes, and simultaneous activation of the line electrodes decreased activation time. Transmural activation was relative to the surface stimulated, and was achieved primarily from endocardial stimulation. Histological evaluation revealed no tissue damage in the cardiac tissue at the end of the studies.

### Activation time according to stimulus strength and line electrode location

#### Single line electrode stimulation

Activation of the cardiac tissue was highly influenced by the location of the line electrodes and the stimulus strength applied. The minimum stimulus magnitude threshold to initiate activation by a single line electrode was 58 ± 11 mA (0.5 ± 0.1 mJ); therefore, 2x thr was 116 ± 21 mA (1 ± 0.4 mJ), and 9x thr was 516 ± 3 mA (15 ± 0.7 mJ). The maximum stimulus magnitude ranged between 6x and 14x thr with an average of 9x (Supplemental Fig. 1A). This maximum was dependent on the limits of the stimulator’s hardware and the initial current requirement to activate the tissue.

#### Multiple line electrode stimulation

The current remained similar when activating multiple lines at the same time. However, the voltage was automatically adjusted to meet the higher energy demand. The energy per channel changed accordingly. When three line electrodes were activated simultaneously, meaning, the whole epicardial or endocardial surface, the energy per channel was on average 1 ± 0.2, 3 ± 0.7 and 27 ± 1 mJ for thr, 2x thr and 9x thr, respectively. For the entire cardiac tissue (all 6 electrodes at the same time), the energy per channel was determined as 1 ± 0.2, 3 ± 0.6 and 39 ± 4 mJ for thr, 2x thr and 9x thr, respectively.

#### Orientation of line electrode(s)

The placement of the line electrode with respect to myocardial fiber orientation did not have a major effect on the current threshold for initial activation or the generation of planar propagating wave fronts. Each line electrode was placed in an apico-basal orientation covering at least 6 cm of the tissue while avoiding surface vasculature or crossing valleys between trabeculae. Consequently, they were not entirely straight and curved on average by 16° (computed between the most bent and straight portions of the line electrode), and ranged from 2 to 35°, meaning lines encountered a mixture of underlying fiber orientations. No correlation was observed between the angle of the line electrode and the initial current threshold (R^2^ = 0.024 and R^2^ = 0.094 for the epicardial and endocardial electrodes, respectively) (Supplemental Fig. 2).

#### Tissue activation versus stimulus strength

**Figure 3:**
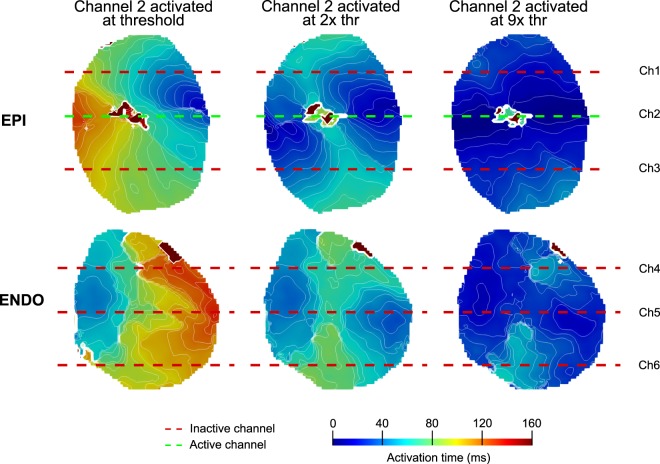
Activation according to stimulation strength. Epicardial and endocardial AT signals during stimulation of channel 2 at thr, 2x and 9x thr. As the stimulation strength increases, linear activation was more prominent around the electrode. Therefore, more tissue was activated in a shorter period of time. Isochrone contour spacing was 8 ms.

**Figure 4:**
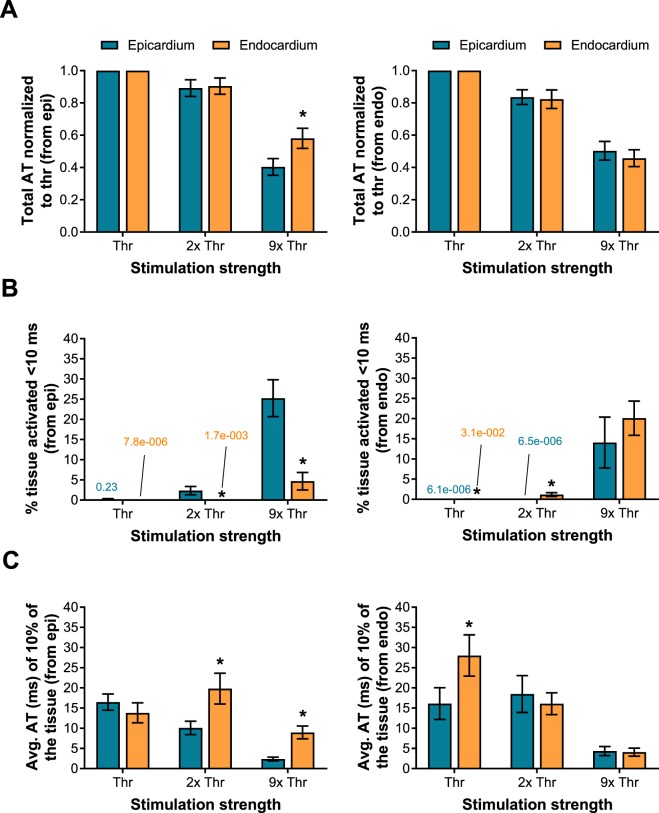
Activation time parameters according to stimulation strength and location with a single line electrode. (**A**) Whole surface stimulation was achieved faster at a stronger strength. No differences (p > 0.05) were observed between epicardial and endocardial surface stimulation at stimulation strength <9x thr. (**B**) The percentage of tissue that was activated within 10 ms from the onset of the stimulation was proportional not just to the stimulation strength, but also to the surface: line activated on the epicardium (left) or line activated on the endocardium (right). (**C**) Similar to (**B**), for stronger stimulation strengths the tissue was activated faster during the early activation phase. Notably, at 2x and 9x thr the epicardium was activated faster than the endocardium if the stimuli originated at the epicardial tissue (p < 0.05), but not when the stimuli originated at the endocardium (p > 0.05).

In general, more tissue was activated in a shorter period of time when the stimulus strength was stronger (Fig. 3). Particularly, the fastest activation from the onset of stimulation (total AT) of the entire map was achieved at 9x thr. On average, when the stimuli originated from the epicardium at 9x thr, the epicardial tissue was activated 60 ± 5% faster than at thr with a single line electrode and 86 ± 4% faster with all three lines. When stimuli originated from the endocardium, the endocardial tissue was activated 54 ± 5% faster at 9x thr than thr with a single line (Fig. 4A) and 80 ± 7% faster with all three lines.

When both surfaces were stimulated simultaneously by all lines, the epicardium was activated 83 ± 4% and the endocardium 80 ± 5% faster than thr, resembling the activation time when each surface was individually stimulated by their respective three lines (p = 0.87 and p = 0.22 for the epicardial and endocardial surfaces, respectively). Furthermore, no statistical difference was observed between the two surfaces (p = 0.34) when individually stimulated by a single line, meaning similar activation times resulted when they were being stimulated independently. However, they were significantly different when single line activation was compared to simultaneous activation of the surfaces from multiple lines at the same time (p = 0.015 and p = 0.01 for the epicardial and endocardial surfaces, respectively).

#### Activation along the line electrode

Planar wave propagation resulting from activation along the entire line was more prominent at 9x thr compared to thr and 2x thr. When examining the early activation of the tissue, it was observed that on average 25 ± 5% of the total epicardial surface was activated within 10 ms from the onset of stimulating an individual line electrode located at the epicardium, and 82 ± 5% from three lines. It was observed that on average 20 ± 4% of the total endocardial surface was activated within 10 ms from the onset of stimulating an individual line electrode located on the endocardial surface and 81 ± 5% from three lines (Fig. 4B).

When both surfaces were stimulated simultaneously with all line electrodes, 84 ± 8% of the epicardium and 83 ± 5% of the endocardium were activated within 10 ms. No statistical differences (p = 0.87 for epicardium and p = 0.74 for endocardium) were noted with respect to individual whole surface stimulation (three electrodes at a time). Statistical differences were also not observed between the two surfaces (p = 0.26) when stimulated individually. However, when compared to single line electrode stimulation they were significantly different when all of the lines located at the epicardium or the endocardium were stimulated at the same time (p = 0.013 and p < 0.0001, respectively).

The initial 10% of the tissue that activated the fastest was around the stimulating electrode at an average of 2 ± 0.5 and 4 ± 1 ms for epicardial and endocardial tissue at 9x thr, respectively (Fig. 4C). When the individual surfaces were stimulated by activating all their electrodes (three at a time), the earliest 10% of total activation time significantly dropped to 0.7 ± 0.3 and 0.3 ± 0.1 ms for the epicardial and endocardial surfaces, respectively (p = 0.01 and p = 0.002). As expected, this was also observed when the entire cardiac tissue was stimulated simultaneously (0.2 ± 0.1 and 0.4 ± 0.1 ms for the epicardium and endocardium, respectively). Statistical differences were not observed between the two surfaces (p = 0.096) when stimulated individually. However, when comparing individual whole surface stimulation (three electrodes at a time) with respect to single electrodes, the activation time from the epicardium and endocardium differed significantly (p = 0.011 and p = 0.002, respectively).

### Tissue activation according to the surface stimulated

**Figure 5:**
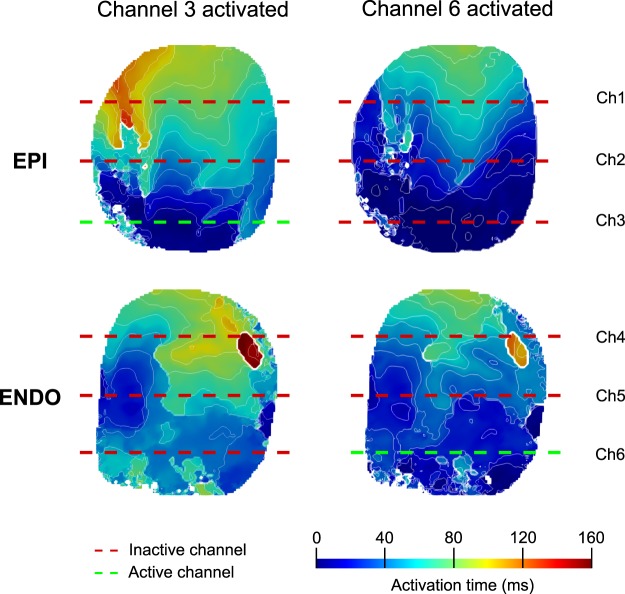
Activation according to the surface stimulated. Epicardial and endocardial AT signals during stimulation of channels 3 and 6 at 9x thr. A more homogeneous activation was seen during epicardial activation compared to endocardial activation, which was evident by the linear activation pattern around the stimulated electrode. Early activation of the stimulated epicardial line was considered faster than the non-stimulated portion of the endocardium (p < 0.05), especially at 2x and 9x thr strength. However, when the endocardial line was stimulated, no differences were evident with respect to the non-stimulated epicardium, especially at 9x thr (p > 0.05). Isochrone contour spacing was 12 ms.

As previously stated, linear activation that produces planar wave propagation was achieved at 9x thr, which correlates with a shorter early activation time around the stimulated electrode with respect to the rest of the tissue. Yet, when one surface was activated by stimulating a line electrode located on that surface, the pattern of early activation on the juxtaposed surface was changed (Fig. 5).

#### Epicardial versus endocardial line electrode stimulation

When the stimulated line electrode was on the epicardium, there was a significant difference in the amount of tissue that was activated within 10 ms (p = 0.001), and also in the average time of activation of the fastest 10% of the tissue (p < 0.0001) with respect to the endocardial surface at 9x thr. In this case, the epicardium activated 21% more tissue and was 6.58 ms faster than the endocardium (Fig. 4B,C). Similar results were obtained when the three line electrodes stimulated the entire epicardium. The epicardium activated 27% more tissue and it was 4 ms faster than the endocardium (p = 0.006 and p = 0.046, respectively).

When the stimuli were administered to the endocardium, no difference (p > 0.05) was observed with respect to the epicardium at 9x thr. An insignificant 6% difference in the amount of tissue activated (p = 0.17) was observed with time differences below the precision of optical recordings (1 ms) (Fig. 4B,C). When the three lines stimulated the entire endocardial tissue, no difference was observed, activating just 5% (p = 0.27) more tissue with time differences also below the precision of optical recordings.

#### Transmural activation

Transmural activation of the endocardial surface was delayed when stimulating the epicardium, but not for the epicardial surface when stimulating the endocardium (6 ± 1 vs 0.5 ± 0.4 ms). This trend was also observed at 2x thr. No difference in the amount of tissue that was activated within 10 ms or the average time of activation of the fastest 10% of the tissue (p = 0.89 and p = 0.14, respectively) was observed between both surfaces when all line electrodes were stimulated simultaneously.

On the contrary, total AT did not significantly differ at 2x thr (p = 0.39 and p = 0.36 from the epicardial and endocardial surfaces, respectively), regardless of the surface or number of line electrodes stimulated. However, it did significantly differ at 9x thr when the stimuli originated from the epicardium (Fig. 4A). When only the epicardial surface was stimulated at 9x thr with a single line electrode, the epicardium was activated 18% faster with respect to the endocardial surface (p < 0.0001), and when it was stimulated using all of the epicardial lines, it was 12% faster (p = 0.038). When only the endocardial layer was stimulated  with a single line electrode, a negligible difference of 5% was observed with respect to the epicardial surface (p = 0.15), which was equivalent to endocardial surface stimulation with all of the endocardial line electrodes (4% faster, p = 0.48). No difference was observed when the entire cardiac tissue was stimulated at the same time with all line electrodes (p = 0.46).

### No tissue damage was observed from line electrode stimulation

**Figure 6:**
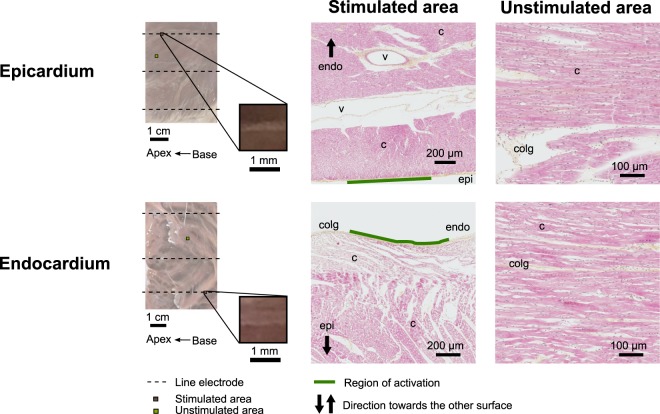
Histological assessment of the cardiac tissue. HES staining was performed to determine if ventricular tissue was affected by the electrical stimulation. Tissue directly under the line electrodes was compared to tissue not in contact with the line electrodes (control). The morphology of the cells was maintained after the stimulation process, and no sign of inflammation was observed, suggesting that cellular damage did not occur. Cardiac fibers were hyper-contracted in some areas of the endocardium, but this was not due to electrode stimulation. *Legend: c* = *cardiomyocytes; v* = *vessel; colg* = *collagen; epi* = *epicardium; endo* = *endocardium*.

HES staining was performed in a subset of the epicardial and endocardial tissue preparations to assess if electrical surface stimulation caused damage in the form of lesions. After the electrodes were removed, grey lines were visible on the tissue’s surface where the electrodes once were (Fig. 6 - left). However, histology revealed no sign of sub-surface coagulation, necrosis, inflammation, or any electrical or mechanical injuries in the tissue surrounding these grey lines (Fig. 6 – middle). Compared to the control case (unstimulated area) for tissue without the grey lines (Fig. 6 - right), line electrodes did not cause any appreciable damage to the cardiac tissue during our experiments, even at the strongest stimulus strength. Hyper-contraction of myocytes was observed on the endocardium (Fig. 6 - middle). However, this was due to fiber bunching from ischemia, not electrical stimulation along the electrode.

## Discussion

We have demonstrated the ability to electrically stimulate wide areas of the epicardial and endocardial surfaces of large mammalian ventricles using line electrodes. Signals were analyzed using a set of variables that encompassed total activation time, as well as early activation parameters in order to quantify linear activation performance. Stimuli of 9x thr activated more tissue in a shorter period of time, particularly in the area surrounding the stimulating electrode to produce a linear activation pattern. Likewise, marked differences were observed between the two cardiac surfaces. During epicardial stimulation, activation time was delayed on the endocardial surface. Whereas, during endocardial stimulation, simultaneous activation with the epicardial surface was noted, suggesting transmural stimulation. Importantly, no tissue or cellular damage was caused by electrode stimulation during our experiments, indicating a potentially safe approach to stimulate wide areas of the heart for cardiac electrotherapies.

### Planar wave front propagation

The importance of planar wave front propagation originating from line electrodes lies in the ability to diminish the influence of tissue inhomogeneities^13^, and to generate planar waves in the presence of heterogeneities. Additionally, uniform linear stimulation increases the possibility of blocking reentrant activation fronts^4^ to help prevent and terminate reentrant sources that underly lethal ventricular arrhythmia. Thus, wide-area surface stimulation with line electrodes could serve as a potential electrotherapy for cardiac defibrillation^14^. The goal would be to achieve activation along the entire line electrode with the lowest current possible.

In our studies, planar wave propagation from a line electrode occurred with stimulation currents as low as 200 mA, or approximately 4x threshold. Though the entire range of stimulation thresholds for complete activation across the line electrode was very broad, up to 14x (Supplemental Fig. 1). This wide-range of thresholds was due to the threshold for initial activation changing considerably from one heart to the next, as well as other factors such as tissue heterogeneity varying under the electrode and the quality of electrical contact between the tissue and the electrode. Therefore, in order to ensure uniform planar waves under all experimental conditions, our data suggest that line electrodes should be stimulated with a minimum of 500 mA delivered at energies ranging from 25 to 39 mJ, which is several orders of magnitude lower than recent low-energy point stimuli studies for ventricular defibrillation^15,16^.

Future studies should focus on how to reduce this upper limit of 500 mA. For example, previous studies^4,17^ suggest that less current may be needed for uniform activation along line electrodes if they are aligned in parallel with the longitudinal fiber orientation of the epicardium. Additionally, electrical current requirements could be reduced by using more durable and higher quality conducting materials (platinum), or by optimizing their contact with the tissue by making adjustments to the line electrode’s diameter and flexibility (braided vs monofilament). See section 4.2 for future studies planned to address these points.

### Wide-area surface stimulation

The activation time of wide areas of the cardiac tissue was highly dependent on stimulus strength and the number of electrodes activated at the same time, impacting both transmural and uniform activation. The stimulation threshold was reached at approximately 0.5–1 mJ, after which depolarization of the cardiac tissue occurred at one or more locations along the line electrodes. Despite regional activation of the cardiac tissue by the electrodes (Fig. 3), the low percentage of tissue activated during the first 10 ms from the onset of stimulation, and the relatively longer AT during the fastest 10% of the tissue (Fig. 4B,C), suggested that non-uniform activation across the entire line was present at threshold stimuli, resembling an activation similar to point electrodes. While at stronger stimulus strengths, planar wave front propagation was achieved over wide areas.

Spatial heterogeneity^18^, anisotropy, the latency of the excitable membrane, and virtual electrode polarization are possible factors that determine performance at various stimulus strengths^19,20^. Similar to other reports^6,7,20–22^, as the stimulus strength was increased, it is possible that a larger virtual cathode was created resulting in simultaneous activation sites and fewer activation delays. Therefore, with currents of at least 500 mA, more tissue was activated within a shorter period of time (Fig. 4 and Supplemental Fig. 1B,C), particularly during the early activation phase, suggesting a more complete capture, and a homogeneous and linear stimulation across the length of the line electrode that would result in planar wave front propagation.

### Endocardial versus epicardial line stimulation for clinical applications

Endocardial wide-area surface stimulation activated more tissue transmurally than epicardial stimulation in a shorter amount of time (Figs 4 and 5), suggesting a more effective electrical conduction from the endocardium to electrically activate a large volume of the heart simultaneously. This conduction pattern is likely attributed to the Purkinje network present within the endocardium, which in pigs covers a large portion of the ventricular cavity as free running fibers that penetrate deep into the subendocardium^23,24^. The Purkinje conduction system is highly specialized and considered to have a conduction velocity approximately 3 times faster than ventricular myocardium, which facilitates a rapid and synchronous activation across the heart^24^. The local effects of the Purkinje conduction system are also believed to be accelerated by the increases in stimulus strength^25^, which would explain the faster activation of the cardiac surfaces at 9x thr (>500 mA) with respect to weaker strengths. It should be noted that pigs, unlike humans, have a fully penetrating Purkinje system^26^. Thus, transmural differences between epicardial and endocardial AT from endocardial stimulation will likely depend on the depth of Purkinje-muscular junctions within the myocardium.

Considering that electrotherapies for complex arrhythmias (VT and VF) often rely on the depolarization of the entire ventricles to close excitable gaps within a few milliseconds^27^, future technologies for defibrillating the ventricles with surface stimulation should target the endocardium. However, further validation in tissue with human-like Purkinje distributions is required^28^. Furthermore, in our studies we were able to stimulate wide-areas of ventricular tissue with energies well below the human pain threshold (100 mJ^29^), which would help avoid several of the inherent risks associated with modern high-energy defibrillation electrotherapies (irreversible myocardial damage, pain, and increased mortality^30^). Although the perceptual pain threshold could vary from one person to another due to a series of factors such as emotional stress and neural activity^29,31^, maintaining energy levels under 100 mJ would have a positive impact not only on patient tolerance to stimuli, but on the device size and endurance as well. Likewise, this technology has the potential to couple with cardiac conformal electronic materials^1,2,32^ to develop new medical devices that fully adjust to the morphology of the heart without imposing any mechanical constraints.

## Conclusions and Future Work

### Conclusions

Electrically stimulating wide areas of the epicardium and endocardium in large mammalian hearts is achievable with line electrodes. Early activation parameters play an important role in determining linear activation performance. Tissue activation was dependent on stimulation strength and the location of the line electrode on either the endocardium or epicardium. In this study, the energies produced per pulse that were necessary for complete activation across a line electrode (<40 mJ) were well below the human pain threshold, and did not damage the tissue. These results support the targeting of cardiac surfaces to painlessly stimulate wide areas of the human heart for safely administering cardiac electrotherapies.

### Future work

Future work will focus on investigating roles of the Purkinje network and cardiac structure on the efficacy of line stimulation. However, changing the position/spacing of the line electrodes, or the properties of the electrodes themselves, is not practical during the *ex-vivo* experiments. This could hinder tissue viability and/or change the initial experimental conditions. As an alternative,computational models developed by the authors^33^ will be employed to investigate the role of Purkinje fiber penetration in transmural activation from the stimulation of the ventricular surfaces, and for different pacing frequencies. These models will also be used to precisely determine how the cardiac fiber orientation (both imaged- and rule-based^34^) alters the initial activation and planar wave propagation, thereby providing a road map for guiding line electrode placement on the ventricular surfaces. Lastly the close resemblance of canine hearts to human heart electrophysiology, particularly in the Purkinje fiber distribution^28^, have shown promising results for investigating cardiac electrotherapies that implement techniques that use lower energies than standard defibrillation practices^8,9,16^. Thus, future work may benefit from studying this animal model, especially for transmural stimulation.

## Limitations

A series of potential limitations need to be considered for the current study. First, the presence of vasculature and fat on the epicardium, as well as trabeculations and Purkinje fibers on the endocardium, limited the position and contact uniformity of the line electrodes. On the epicardium, a risk of partial ischemia was noticed due to possible vasculature occlusion from tightly fitted line electrodes, and adipose tissue had the potential to diminish electrical conduction. Whereas, the irregular endocardial surface, due to trabeculae and papillary muscles, made it challenging to have continuous electrical contact between the line electrodes and the myocardium. Second, there was not a rigorous method put in place to assess full electrical contact along the line electrodes. For this study, electrical contact was assessed by calculating impedance at the threshold for initial activation. From our experience, if impedance was above 100 Ω and/or the delivered electrical current was above 100 mA, there was a higher chance of poor electrical contact. This was verified by visual inspection along the line electrode for gaps in electrical contact with the tissue. Third, the number of line electrodes available to place on the cardiac surfaces was limited by the number of isolated channels in the constant current generator, where the maximum was 10 for the specific stimulator used for this study. Moreover, the position of the line electrodes when assessing the activation maps is based on the analysis of early activation parameters and raw (pre-processed) optical mapping images. Therefore, analyzing linear activation with respect to the location of the electrode is a close approximation, but not an exact representation of the position across the entire line.

## Methods

### Heart preparation

Animal protocols were approved by the local ethical committee (CEEA50) at the University of Bordeaux. All experiments were performed in accordance with the guidelines from Directive 2010/63/EU of the European Parliament on the protection of animals used for scientific purposes. Hearts were obtained from age (3 months) matched large white pigs (n = 7) weighing 40 to 50 Kg. Pigs were pre-medicated with ketamine (20 mg/kg IM), acepromazine (0.1 mg/kg IM) and Buprenorphine (9 μg/kg IM). Anesthesia was induced by propofol (1 mg/kg IV) and maintained under isoflurane, 1.5–3%, in air/O_2_ (50/50%) after intratracheal intubation. Pigs were heparinized (200 UI/kg IV) and then euthanized by intravenous injection with pentobarbital (80 mg/kg IV). Once cardiac arrest was confirmed, hearts were quickly excised, cannulated by the aorta, and rinsed with an ice-cold cardioplegic solution containing, in mM: 110 NaCl; 10 NaHCO_3_; 16 MgCl_2_; 10 Glucose; 16 KCl; and 1.2 CaCl_2_.

A left ventricular (LV) wedge preparation from these hearts was used in our experiments. Briefly, the left coronary ostium was quickly isolated and cannulated. To maintain pressure, any major arterial leaks were ligated with silk suture. Once hearts were cannulated, they were placed in a constant flow (30 mL/min) perfusion system with oxygenated (95% O_2_, 5% CO_2_) bicarbonate-buffered solution containing, in mM: 130 NaCl; 24 NaHCO_3_; 1.2 NaH_2_PO_4_; 1 MgCl_2_; 5.6 Glucose; 4 KCl; and 1.8 CaCl_2_. Constant temperature was maintained in the system at 37 °C.

### Optical mapping

To suppress mechanical activity and to minimize motion artifacts, the electro-mechanical uncoupler, blebbistatin (10 µM), was administered. Preparations were then perfused with the voltage-sensitive dye Di-4-ANEPPS (5 µM) to detect changes in membrane potential during optical action potential (OAP) recordings. Tissue was illuminated by four LEDs (530 nm, CAIRN Research) and the emitted signal was band-pass filtered (650 ± 40 nm) and recorded using two CMOS cameras (MiCAM Ultima; SciMedia, Ca) that imaged the epicardial and endocardial surfaces with fine temporal and spatial resolution (1000 frames/sec, 100 × 100 pixels).

### Cardiac stimulation protocol

Three 8 cm copper line electrodes, each 1 mm in diameter, were placed 2–3 cm apart on each side of the wedge (epicardium and endocardium) (Fig. 1A). These linear electrodes were stimulated in unipolar mode using a constant current pulse generator (D343; Digitimer, UK) with 10 independent current sources that permitted individual line activation at 2.5 Hz (just faster than normal 60–120 bpm heart rates of a pig during sinus rhythm), 2 ms pulse width (Fig. 1B), and at different output currents ranging from 46 to 530 mA per pulse corresponding to threshold (thr, minimum current necessary to pace the heart), 2x thr, and 9x thr (equipment limit). Output voltage and current from individual channels were measured using the built in meter module (D335; Digitimer, UK) located at the constant current pulse generator. Electrical energy (E) was calculated as E = V*I*t, where V was the voltage, I the current, and t the duration of the pulse width. The ground electrode was an 8 cm long, 1.13 mm in diameter annealed copper wire placed as far away as possible at the bottom of the perfusion chamber, which was a distance of 1.5–2 cm from the apical region of the tissue. The ground was centered between the epicardial and endocardial surfaces to ensure a relatively uniform effect on the unipolar electrodes. Responses to line stimulation were monitored (LabChart, AD Instruments) using a bath ECG.

### Data analysis

Optical mapping data were processed using analysis functions based on the PV-WAVE programming platform (Rogue Wave Software, Co). OAPs were filtered by a first-order low-pass Butterworth filter with a cutoff frequency at 120 Hz and by a 3 × 3 pixel spatial filter. Activation time (AT) was computed as the dV/dt_max_ of OAPs and used to build the activation maps. Activation maps were plotted using ParaView 5.4.1 (Kitware, NY). From these maps, a custom Matlab (MathWorks, Ma) program was used to analyze several parameters to assess the effects of the stimulation lines on AT: total AT; percentage of tissue that was activated within 10 ms; and average time to activate 10% of the sample (Figs 1C and 2).

Total AT was used to assess the time it takes to activate the entire tissue from the onset of stimulation (maximum-minimum AT). Since total AT was dependent upon the position of the line electrodes relative to the imaged field of view, total AT was also normalized for each electrode based on the total AT at thr (respectively), and then averaged. In general, total AT is an indication of overall activation performance and not a full representation of individual linear activation. Consequently, in order to measure early AT, and therefore to evaluate homogeneity of stimulation along the electrode line, two more variables were used.

The first early activation parameter computes the percentage of tissue that is activated within a 10 ms window from the onset of the stimulation. This window was chosen based on preliminary results. Within 10 ms and when all the line electrodes are activated simultaneously at 9x thr, up to 92% of the entire tissue is activated. Also, 10 ms is within the time frame for total AT. The second variable quantifies the average time to activate the first 10% of the sample from the onset of stimulation. This is considered the immediate area surrounding a line electrode that has been activated.

### Assessment of cellular damage

A subset of LV wedges (n = 2) were fixed in 4% paraformaldehyde, and transmural slices were prepared for Hematoxylin-Eosin-Saffron (HES) staining (transmural sections of 6 µm). This allowed verification of tissue damage resulting from line stimulation. HES stains cardiomyocyte cytoplasm pink, the cell nucleus black, and collagen fibers orange. Slices were examined at 20x magnification under an Axio Scan.Z1 (ZEISS, DE) slide scanner. Images were plotted using Zen Lite (ZEISS, DE).

### Statistical analysis

Statistical analysis was performed using GraphPad Prim 7.04 (GraphPad Sotware, Ca). Data were presented as mean ± standard error of the mean. A paired t-test was used to compare between paired data sets. Significance was defined as p < 0.05. A Shapiro-Wilk test was performed to determine normality in the data.

## Supplementary information

Supplementary Figures

## Data Availability

The datasets generated during and/or analyzed during the current study are available from the corresponding author upon reasonable request.
