# Supplementary material for: Wide-area low-energy surface stimulation of large mammalian ventricular tissue
Source: Sci Rep. 2019 Nov 1;9:15863. doi: 10.1038/s41598-019-51364-w (PMC6825186; doi:10.1038/s41598-019-51364-w)
Supplement: Supplementary file 1 — Supplementary Figures [file 41598_2019_51364_MOESM1_ESM.pdf]

Supplementary material: Wide-area low-energy surface stimulation of large mammalian ventricular tissue

Angel Moreno [1,2]**,** Richard D. Walton [1,3,4], Marion Constantin [1,3,4], Olivier Bernus [1,3,4], Edward J. Vigmond [1,2], Jason D. Bayer* [1,2]

L'Institut de Rythmologie et Modélisation Cardiaque (LIRYC). Hôpital Xavier Arnozan, Avenue du Haut Lévêque, 33604 Pessac, France

[1] IHU-LIRYC, Electrophysiology and Heart Modeling Institute, Fondation Bordeaux Université. Pessac- Bordeaux, France.
[2] Centre National De La Recherche Scientifique, Institut de Mathématiques de Bordeaux, UMR5251. Bordeaux, France.
[3] Centre de Recherche Cardio-Thoracique de Bordeaux, Université de Bordeaux, U1045. Bordeaux, France.
[4] INSERM, Centre de recherche Cardio-Thoracique de Bordeaux, U1045. Bordeaux, France.

Corresponding author:

Jason D. Bayer

Address: L'Institut de Rythmologie et Modélisation Cardiaque (LIRYC), Hôpital Xavier Arnozan, Avenue du Haut Lévêque, 33604 Pessac, France
Phone: +33-5 35 38 19 65
Email: Jason.Bayer@ihu-liryc.fr

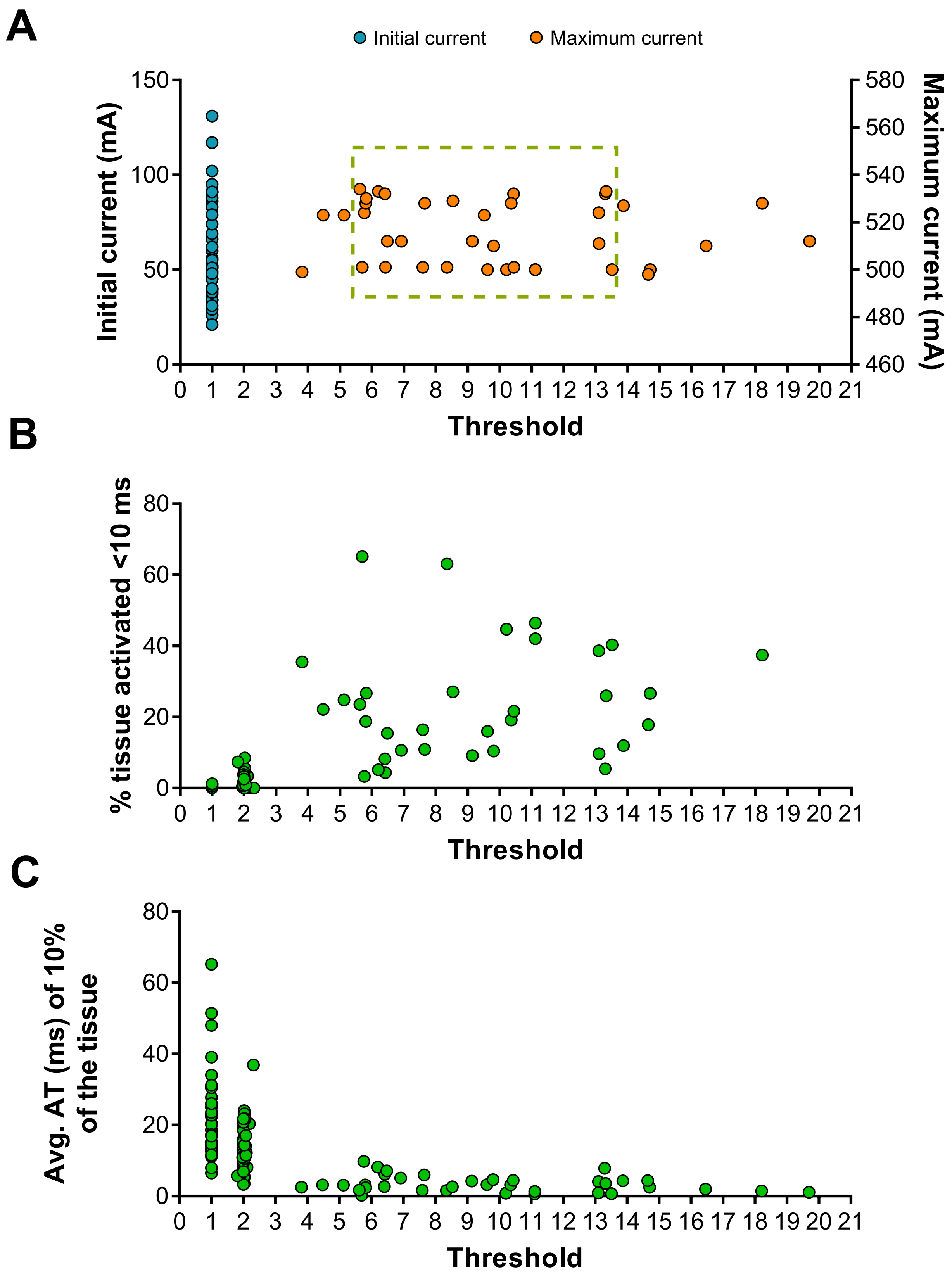

Supplemental figure 1. Thresholds according to initial current for activation and to early activation parameters. (A) Current thresholds for initial activation and at maximum current do not necessarily have to be functions of one another. Tissue heterogeneities, quality of the electrical contact, and tissue properties are some of the reasons for the initial variation of electrical current requirement.

(B) More tissue was activated as the energy increased, predominantly between 6x and 14x threshold, at which uniform and linear activation was reached. (C) Similarly, as the energy delivery increased, the tissue surrounding the line electrode was activated in a shorter time.

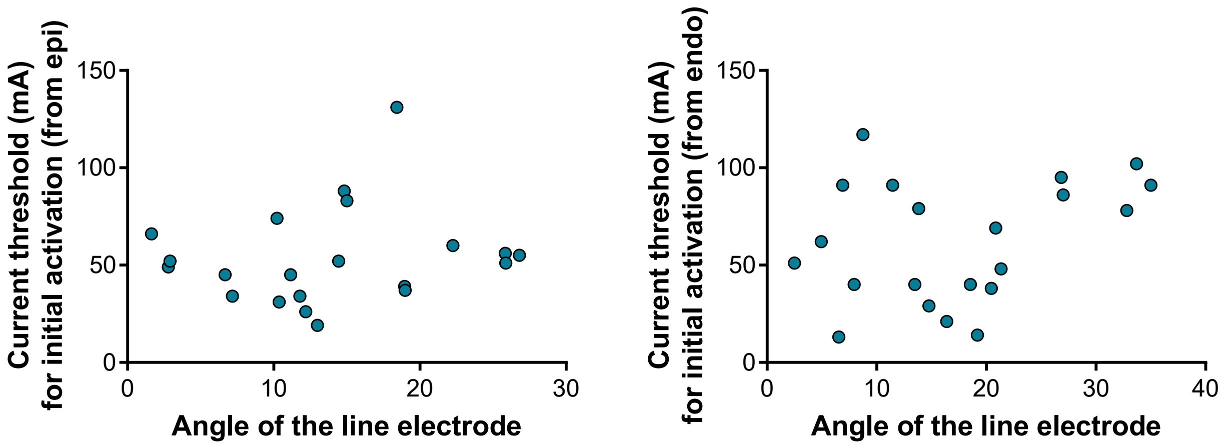

Supplemental figure 2. Current threshold for initial activation according to the angle at which the line is placed. The position of the lines was adjusted to the morphology of the cardiac tissue in order to avoid vasculature constrictions and to improve electrical contact. Despite the overall position of the line, there was no relation between its angle and the initial current requirement for activation regardless of the surface.
